# Supplementary material for: Bone impact after two years of low-dose oral contraceptive use during adolescence
Source: PLoS One. 2023 Jun 8;18(6):e0285885. doi: 10.1371/journal.pone.0285885 (PMC10249826; doi:10.1371/journal.pone.0285885)
Supplement: S1 Table — (DOCX) [file pone.0285885.s001.docx]

**S1 Table. Multiple linear regression for the comparison of the evolution of the variables between the groups adjusted by basal bone age, BMI, and Total body BMD.**

| **Outcome: Lumbar BMD_0a24** | **b** |  | **IC95%** |  | ***p*** |
| --- | --- | --- | --- | --- | --- |
| Intercept | 0.25 | -0.01 |  | 0.50 | *0.062* |
| COC2 | -0.05 | -0.11 |  | 0.01 | *0.089* |
| COC1  Controls | -0.02  0a | -0.09 |  | 0.05 | *0.602* |
| Bone age 0 | -0.02 | -0.03 |  | -0.01 | *0.006* |
| BMI 0 | 0.00 | 0.00 |  | 0.01 | *0.329* |
| Total body BMD 0 | 0.04 | -0.24 |  | 0.31 | *0.793* |
| p-Shapiro Wilk for residuals = 0.584 ; weak heterocedasticity |  |  |  |  |  |
| **Outcome: Lumbar BMC_0a24** | **b** |  | **IC95%** |  | *p* |
| Intercept | 31.88 | 13.60 |  | 50.16 | *0.001* |
| COC2 | -5.61 | -9.61 |  | -1.61 | ***0.006*** |
| COC1  Controls | -3.60  0a | -8.58 |  | 1.38 | *0.157* |
| Bone age 0 | -1.46 | -2.39 | -0.52 | | *0.002* |
| BMI 0 | 0.02 | -0.37 | 0.41 | | *0.932* |
| Total body BMD 0 | -3.18 | -22.61 | 16.25 | | *0.749* |
| p-Shapiro Wilk for residuals = 0.821 ; non-heterocedasticity |  |  |  | |  |

| **Outcome: Total body BMD _0a24** | **b** | **I** | **C95%** |  | *p* |
| --- | --- | --- | --- | --- | --- |
| Intercept | 0.33 | 0.17 |  | 0.49 | *0.000* |
| COC2 | -0.10 | -0.14 |  | -0.07 | ***0.000*** |
| COC1  Controls | -0.11  0a | -0.16 |  | -0.07 | ***0.000*** |
| Bone age 0 | 0.00 | -0.01 |  | 0.01 | *0.632* |
| BMI 0 | 0.00 | 0.00 |  | 0.00 | *0.578* |
| Total body BMD 0 | -0.15 | -0.32 |  | 0.02 | *0.081* |
| p-Shapiro Wilk for residuals = 0.141 ; non-heterocedasticity |  |  |  |  |  |
| **Outcome: Total body BMC_0a24** | **b** | **I** | **C95%** |  | *P* |
| Intercept | 1,026.47 | 653.18 | 1,399.75 | | *0.000* |
| COC2 | -315.41 | -395.82 | -234.99 | | ***0.000*** |
| COC1 | -322.39 | -421.94 | -222.83 | | ***0.000*** |
| Controls | 0a |  |  |  |  |
| Bone age 0 | -33.43 | -52.79 | -14.07 | | *0.001* |
| BMI 0 | 1.21 | -6.79 | 9.22 | | *0.766* |
| Total body BMD 0 | -170.27 | -569.41 | 228.88 | | *0.403* |
| p-Shapiro Wilk for residuals = 0.148 ; non-heterocedasticity |  |  |  | |  |
| **Outcome: Subtotal BMD_0a24** | **b** | **I** | **C95%** | | *P* |
| Intercept | 0.17 | 0.06 | 0.28 | | *0.002* |
| COC2 | -0.02 | -0.05 | 0.00 | | *0.054* |
| COC1 | -0.03 | -0.06 | -0.01 | | ***0.017*** |
| Controls | 0a |  |  |  |  |
| Bone age 0 | -0.01 | -0.01 |  | 0.00 | *0.052* |
| BMI 0 | 0.00 | 0.00 |  | 0.00 | *0.340* |
| Total body BMD 0 | -0.07 | -0.18 |  | 0.05 | *0.239* |
| p-Shapiro Wilk for residuals = 0.282 ; non-heterocedasticity |  |  |  |  |  |
| **Outcome: Subtotal BMC_0a24** | **b** | **I** | **C95%** |  | *P* |
| Intercept | 347.24 | -122.83 | 817.31 | | *0.148* |
| COC2 | -101.88 | -203.14 | -0.61 | | ***0.049*** |
| COC1 | -77.97 | -203.34 | 47.39 | | *0.223* |
| Controls | 0a |  |  |  |  |
| Bone age 0 | -16.16 | -40.53 | 8.22 | | *0.194* |
| BMI 0 | -0.24 | -10.33 | 9.84 | | *0.962* |
| Total body BMD 0 | 21.07 | -481.56 | 523.69 | | *0.935* |
| p-Shapiro Wilk for residuals = 0.006 ; non-heterocedasticity |  |  |  | |  |

| **Outcome: Fat mass_0a24** | **b** | **IC95%** | | | | *P* |
| --- | --- | --- | --- | --- | --- | --- |
| Intercept | 1,172.91 | -2,2437.17 | | 2,4782.99 | | *0.922* |
| COC2 | -61.80 | -5,013.56 | | 4,889.96 | | *0.980* |
| COC1  Controls | -661.70  0a | -6,564.09 | | 5,240.69 | | *0.826* |
| Bone age 0 | -839.89 | -1,972.13 | | 292.34 | | *0.146* |
| BMI 0 | 139.55 | -322.81 | | 601.90 | | *0.554* |
| Total body BMD 0 | 12,098.85 | -12,509.96 | | 36,707.66 | | *0.335* |
| p-Shapiro Wilk for residuals < 0.001 ; non-heterocedasticity |  |  | |  | |  |
| **Outcome: BAP_0a24** | **b** | **IC95%** | | | | ***P*** |
| Intercept | -216.86 | -293.35 | | | -140.37 | *0.000* |
| COC2 | -6.08 | -21.64 | | | 9.49 | *0.444* |
| COC1 | -0.71 | -19.08 | | | 17.67 | *0.940* |
| Controls | 0a |  | |  |  |  |
| Bone age 0 | 8.02 | 4.35 | 11.68 | | | *0.000* |
| BMI 0 | 1.11 | -0.39 | 2.62 | | | *0.147* |
| Total body BMD 0 | 47.32 | -32.17 | 126.82 | | | *0.243* |
| p-Shapiro Wilk for residuals = 0.314 ; non-heterocedasticity |  |  |  | | |  |
| **Outcome: Osteocalcin_0a24** | **b** |  | **IC95%** | | | *P* |
| Intercept | -36.23 | -66.18 | -6.28 | | | *0.018* |
| COC2 | -0.90 | -6.88 | 5.09 | | | *0.768* |
| COC1 | 0.24 | -6.65 | 7.14 | | | *0.945* |
| Controls | 0a |  |  |  |  |  |
| Bone age 0 | 2.58 | 1.14 | 4.02 | | | *0.000* |
| BMI 0 | 0.20 | -0.38 | 0.78 | | | *0.495* |
| Total body BMD 0 | -11.98 | -43.04 | 19.07 | | | *0.450* |
| p-Shapiro Wilk for residuals = 0.693 ; non-heterocedasticity |  |  |  | | |  |
| *Note:* Controls: adolescents who did not use oral contraceptives. |  |  |  | | |  |

COC1: adolescents receiving an oral contraceptive containing 20 μg EE/150 μg desogestrel.

COC2: adolescents receiving an oral contraceptive containing 30 μg EE/3 mg drospirenone.

BMI: Body mass index; BMD: Bone mineral density; BMC: Bone mineral content;

BAP: Bone alkaline phosphatase;

a: reference value for estimate b in the control group
