# Supplementary material for: Bone impact after two years of low-dose oral contraceptive use during adolescence
Source: PLoS One. 2023 Jun 8;18(6):e0285885. doi: 10.1371/journal.pone.0285885 (PMC10249826; doi:10.1371/journal.pone.0285885)
Supplement: S2 Table — (DOCX) [file pone.0285885.s002.docx]

**S2Table. Comparison of anthropometric and densitometric variables and bone formation markers at baseline between adolescents receiving low-dose oral contraceptives and the control group for complete cases.**

|  | **Controls (n=19)** | | **COC1 (n=23)** | | **COC2 (n= 34)** | |  |  |
| --- | --- | --- | --- | --- | --- | --- | --- | --- |
|  | **Mean** | **SD** | **Mean** | **SD** | **Mean** | **SD** | ***P Value*** |  |
| Age(years) | 15.4 | 2.0 | 15.9 | 1.7 | 15.5 | 1.4 | 0.545 |  |
| Bone age (years) | 15.8 | 1.9 | 16.1 | 1.2 | 16.3 | 1.1 | 0.578 |  |
| Weight (kg) | 55.0 | 8.4 | 52.7 | 8.0 | 54.2 | 8.3 | 0.683 |  |
| Height (cm) | 161.2 | 5.0 | 159.9 | 6.5 | 157.7 | 6.9 | 0.142 |  |
| BMI (kg/m^2^) | 21.0 | 3.0 | 20.6 | 2.6 | 21.7 | 2.8 | 0.346 |  |
| Z-score for BMI | 0.2 | 0.8 | 0.0 | 0.8 | 0.4 | 0.7 | 0.138 |  |
| BMI (percentile) | 55.3 | 25.5 | 49.2 | 26.1 | 62.5 | 22.4 | 0.154 |  |
| Lumbar BMD (g/cm^2^) | 0.898 | 0.087 | 0.905 | 0.063 | 0.948 | 0.108 | 0.138 |  |
| Lumbar BMC (g) | 47.08 | 9.02 | 48.03 | 5.17 | 50.32 | 8.14 | 0.333 |  |
| Z-score for lumbar | -0.4 | 0.8 | -0.6 | 0.8 | -0.1 | 1.0 | 0.169 |  |
| Total body BMD (g/cm^2^) | 1.130 ^a^ | 0.073 | 0.974 ^b^ | 0.053 | 1.016 ^b^ | 0.070 | **0.000** |  |
| Total body BMC (g) | 1,982.04 | 277.09 | 1,783.17 | 205.59 | 1,815.40 | 249.53 | **0.037** |  |
| Z-score for total body | 1.4 ^a^ | 0.9 | -1.1 ^b^ | 0.6 | -0.4 ^c^ | 0.9 | **0.000** |  |
| Subtotal BMD (g/cm^2^) | 0.911 ^a^ | 0.051 | 0.852 ^b^ | 0.049 | 0.890 ^ab^ | 0.062 | **0.013** |  |
| Subtotal BMC (g) | 1,366.27 | 214.76 | 1,364.67 | 165.25 | 1,395.26 | 209.01 | 0.839 |  |
| Fat mass (g) | 18,666.3 | 4330.4 | 17,722.1 | 6610.5 | 17,903.0 | 5262.2 | 0.851 |  |
| Lean mass (g) | 34,590.2 | 4,822.7 | 36,724.1 | 4,442.7 | 35,093.6 | 7953.5 | 0.625 |  |
| Total body fat (%) | 33.8 | 4.3 | 29.9 | 4.0 | 31.6 | 5.1 | 0.058 |  |
| BAP (U/L) | 47.07 | 23.85 | 56.14 | 32.07 | 44.26 | 19.68 | 0.322 |  |
| Osteocalcin (ng/mL) | 16.66 a | 8.10 | 9.32 b | 5.15 | 10.97 b | 6.25 | **0.003** |  |
|  |  |  |  |  |  |  |  |  |

*Note:* Controls: adolescents who did not use oral contraceptives.

COC1: adolescents receiving an oral contraceptive containing 20 μg EE/150 μg desogestrel.

COC2: adolescents receiving an oral contraceptive containing 30 μg EE/3 mg drospirenone.

BMI: Body mass index; BMD: Bone mineral density; BMC: Bone mineral content; BAP: Bone alkaline phosphatase;

ANOVA model with fixed effects followed by Bonferroni for comparison of means between the three groups.

Different lowercase letters indicate significant differences between the three groups (p<0.05).
