# Supplementary material for: Bone impact after two years of low-dose oral contraceptive use during adolescence
Source: PLoS One. 2023 Jun 8;18(6):e0285885. doi: 10.1371/journal.pone.0285885 (PMC10249826; doi:10.1371/journal.pone.0285885)
Supplement: S3 Table — (DOCX) [file pone.0285885.s003.docx]

**S3 Table . Comparison of the variation in mean absolute values of the anthropometric and densitometric variables and bone formation markers between adolescents receiving low-dose oral contraceptives and the control group followed up for 24 months**

|  |  | **Controls (n=19)** | | | |  | | **COC1 (n=23)** | | | |  | | **COC2 (n=34)** | | | |  | |
| --- | --- | --- | --- | --- | --- | --- | --- | --- | --- | --- | --- | --- | --- | --- | --- | --- | --- | --- | --- |
|  |  | **Mean** | **SD** | **min** | **max** |  | **Mean** | | **SD** | **Min** | **Max** |  | **Mean** | | **SD** | **min** | **Max** | ***P*** |  |
|  | Bone age (years) | 1.32 | 1.15 | 0.00 | 3.00 |  | 0.67 | | 0.52 | 0.00 | 1.00 |  | 0.97 | | 0.85 | 0.00 | 2.00 | *0.367* |  |
|  | Weight (kg) | 4.18 | 4.67 | -5.00 | 11.00 |  | 3.82 | | 5.28 | -3.30 | 16.60 |  | 2.75 | | 3.52 | -4.50 | 11.40 | *0.473* |  |
|  | Height (cm) | 0.74 | 0.82 | 0.00 | 3.00 |  | 0.8 | | 1.39 | -2.00 | 4.00 |  | 1.06 | | 0.99 | -0.50 | 4.00 | *0.516* |  |
|  | BMI (kg/m^2^) | 1.42 | 1.67 | -2.03 | 3.61 |  | 1.08 | | 1.92 | -1.88 | 5.57 |  | 0.67 | | 1.49 | -2.31 | 3.83 | *0.274* |  |
|  | Z-score for BMI | 0.13 | 0.49 | -0.74 | 0.76 |  | 0.00 | | 0.50 | -0.85 | 0.59 |  | -0.10 | | 0.39 | -0.87 | 0.78 | *0.219* |  |
|  | BMI (percentile) | 4.04 | 17.72 | -26.84 | 28.28 |  | -0.37 | | 16.00 | -32.5 | 22.56 |  | -3.71 | | 13.6 | -33.17 | 28.72 | *0.243* |  |
|  | Lumbar BMD (g/cm^2^) | 0.051 ^a^ | 0.041 | -0.026 | 0.113 |  | 0.019^ab^ | | 0.048 | -0.070 | 0.095 |  | -0.012 ^b^ | | 0.063 | -0.159 | 0.118 | ***0.001*** |  |
|  | Lumbar BMC (g) | 4.85 ^a^ | 4.86 | -3.86 | 12.64 |  | 2.15^ab^ | | 3.10 | -3.17 | 6.97 |  | -0.43 ^b^ | | 4.28 | -9.76 | 11.56 | ***0.001*** |  |
|  | Z-score for lumbar | 0.0 ^a^ | 0.5 | -0.6 | 1.0 |  | -0.2^ab^ | | 0.5 | -0.8 | 0.8 |  | -0.6 ^b^ | | 0.6 | -1.8 | 0.7 | ***0.002*** |  |
|  | Total body BMD (g/cm^2^) | 0.095 ^a^ | 0.039 | 0.004 | 0.160 |  | 0.018 ^b^ | | 0.024 | -0.034 | 0.050 |  | 0.021 ^b^ | | 0.040 | -0.041 | 0.148 | ***0.000*** |  |
|  | Total body BMC (g) | 294.80 ^a^ | 151.96 | 38.36 | 615.62 |  | 27.90 ^b^ | | 49.74 | -39.10 | 106.73 |  | 21.11 ^b^ | | 60.21 | -90.62 | 126.04 | ***0.000*** |  |
|  | Z-score for total body | 0.7 ^a^ | 0.4 | -0.3 | 1.2 |  | 0.1 ^b^ | | 0.3 | -0.7 | 0.5 |  | -0.4 ^b^ | | 0.5 | -1.2 | 0.6 | ***0.000*** |  |
|  | Subtotal BMD (g/cm^2^) | 0.026 | 0.028 | -0.017 | 0.073 |  | 0.005 | | 0.016 | -0.033 | 0.028 |  | 0.014 | | 0.025 | -0.031 | 0.080 | *0.056* |  |
|  | Subtotal BMC (g) | 100.83^a^ | 116.36 | -54.42 | 334.67 |  | 21.56^ab^ | | 88.67 | -59.66 | 270.12 |  | -1.47 ^b^ | | 86.08 | -321.6 | 126.38 | ***0.005*** |  |
|  | Fat mass (g) | 3,640.35 | 3,702.59 | -3,631.80 | 10,552.50 |  | 1,941.75 | | 7,329.51 | -18,075.10 | 11,593.90 |  | 2,634.48 | | 2,535.80 | -1,580.70 | 9,569.10 | *0.566* |  |
|  | Lean mass (g) | -1,048.01 | 3,099.56 | -5,735.70 | 4,462.80 |  | 968.08 | | 2,710.00 | -3,446.40 | 5,384.40 |  | 1,622.05 | | 5,550.62 | -2,950.50 | 24,282.61 | *0.145* |  |
|  | Total body fat (%) | 4.43 | 4.76 | -3.30 | 13.70 |  | 3.08 | | 3.22 | -2.30 | 8.50 |  | 2.58 | | 2.90 | -4.20 | 8.30 | *0.293* |  |
|  | BAP (U/L) | -17.02 | 19.09 | -65.18 | 10.67 |  | -17.79 | | 25.67 | -79.52 | 10.35 |  | -12.36 | | 11.94 | -39.68 | 3.26 | *0.686* |  |
|  | Osteocalcin (ng/mL) | -2.70 | 9.26 | -19.87 | 11.82 |  | -3.02 | | 6.03 | -13.61 | 11.54 |  | -1.97 | | 4.76 | -10.61 | 5.80 | *0.909* |  |

*Note:* Controls: adolescents who did not use oral contraceptives.

COC1: adolescents receiving an oral contraceptive containing 20 μg EE/150 μg desogestrel.

COC2: adolescents receiving an oral contraceptive containing 30 μg EE/3 mg drospirenone.

BMI: Body mass index

BMD: Bone mineral density

BMC: Bone mineral content

BAP: Bone alkaline phosphatase

ANOVA for comparison of means between the three groups.

Different lowercase letters indicate significant differences between the three groups (p<0.05). Bonferroni test for multiple comparisons between the three groups.
