# Supplementary material for: Bone impact after two years of low-dose oral contraceptive use during adolescence
Source: PLoS One. 2023 Jun 8;18(6):e0285885. doi: 10.1371/journal.pone.0285885 (PMC10249826; doi:10.1371/journal.pone.0285885)
Supplement: S1 File — (PDF) [file pone.0285885.s004.pdf]

Trial Protocol

English Version

Bone impact after two years of low-dose oral contraceptive use during adolescence

Objective: The present study was designed to evaluate bone metabolism in two groups of healthy adolescents using combined oral contraceptives (COC).

This was a non-randomized clinical trial that included healthy adolescent girls aged 12 to 20 incomplete years, recruited from 2014 to 2020. The study is registered under clinical trial registration number RBR-5h9b3c with the title “Bone mineral density in adolescents using combined oral contraceptives”.

The girls were volunteers seen on an outpatient basis and classified as Tanner stages B4 or B5, who already had their first menstruation and who had regular menstrual cycles.

Patients without an indication for use of a contraceptive method because they had no active sexual life were allocated to the control group. For girls with an indication, all contraceptive methods appropriate for this age group were presented by the health professionals and those who chose to use COC were included in the study. The COC methods defined were combinations of 20 µg Ethinylestradiol (EE)/150 µg Desogestrel (COC1 group) and 30 µg EE/3 mg Drospirenone (COC2 group).

All participants were advised and encouraged to use dual protection (male condom concomitantly with the contraceptive method) in order to prevent sexually transmitted

infections. In addition, all the girls were healthy, non-smokers and non-alcohol drinkers, who did not use illicit drugs or medications such as anticonvulsants, anticoagulants, antiretroviral agents, antacids containing aluminum, corticosteroids, or calcium or iron supplements that could interfere with bone mass gain. The participants did not practice sports outside the school and the participation in school sports did not exceed 2 h per week. Adolescents with chronic renal, gastrointestinal, or endocrine diseases, such as diabetes mellitus, early or late puberty, or polycystic ovary syndromes and those with a history of oral contraceptive use or pregnancy were not eligible.

Written informed consent was obtained from all individual participants included in the study or their legal representative for authorization and participation in the study. The project was submitted to the Research Ethics Committee of the Botucatu School of Medicine, São Paulo, Brazil (ethical clearance certificate number 52928416.6.0000.5411) and an amendment was approved by Plataforma Brasil under number 2.766.807.

Anthropometric data were collected and secondary sexual characteristics were evaluated by visual inspection of the breasts and pubic hair and classified according to the Tanner criteria [2]. The adolescents participating in the study were healthy and had a height and body mass index (BMI) between the 5th and 95th percentile for each age group according to the Centers for Disease Control and Prevention growth charts [15]. Bone age (BA) for evaluation of the degree of skeletal maturation was obtained from all adolescents using the method of Greulich & Pyle. The data were interpreted by a single trained evaluator who was unaware of the group to which the adolescente belonged (evaluator blinding).

Bone mineral density (BMD) was evaluated in all adolescents (controls and COC users) at the time of inclusion in the study and after 12 and 24 months of follow- up by dual-energy X-ray absorptiometry (DXA), using a Hologic QDR 4500 Discovery A densitometer (Hologic Inc., Bedford, MA). Lumbar spine (L1-L4) and total and subtotal (without head segment) BMD measurements were obtained [16]. All assessments were performed by a

trained professional, who was unaware whether or not the adolescent used a COC (evaluator blinding).

Blood samples were collected by venipuncture, in the morning after a 10-hour fast, and centrifuged for 15 min at 1,500 g for the separation of serum. The samples were stored at -70oC until the time of analysis of the biomarkers [bone alkaline phosphatase (BAP) and osteocalcin]. Osteocalcin and BAP were measured using the MicroVue Enzyme Immunoassay (EIA) (Quidel®, San Diego, CA, USA). This immunoassay is a competitive ELISA test that quantifies only intact osteocalcin (ng/mL) as an indicator of bone turnover and does not detect fragments of reabsorbed bone tissue. The intra- and inter-assay coefficients of variation obtained as a measure of precision of the assay ranged from 5 to 10%, as recommended by the manufacturer. For BAP, expressed in U/L, the intra-assay coefficient of variation ranged from 4 to 6% and the inter-assay coefficient of variation ranged from 5 to 8%.

All adolescents were evaluated at intervals of 3 months during the proposed follow-up period of two years. On this occasion, the continuity of use of the prescribed COC was analyzed, as well as the permanence of the adolescent in the study according to the strict criteria proposed for their inclusion. In the case of adolescentes who did not appear on the scheduled day for densitometry or blood collection, new appointments were offered close to the days defined for the examinations. If the adolescents did not show up on any of the three scheduled days for the examinations at each proposed time point, or if the results obtained at 12 or 24 months were incomplete, the adolescent was included in the statistical analyses up to her participation in the follow-up. The same care was provided to all adolescents who continued in the follow-up for the proposed period, according to the guidelines of the health services they attended.

Statistical Analysis

First, the homogeneity of the groups was verified. There was no significant violation of the theoretical assumptions of normality of the residues (through the Shapiro-Wilk test and histograms) and homoscedasticity (through the Levene test and dispersion between residuals and predictions of the models), corroborating the adopted models.

Comparisons between groups regarding anthropometric, densitometric, and bone marker variables at baseline and after 24 months involving all selected participants were performed using the ANOVA model with fixed effects, followed by the Bonferroni test for multiple comparisons.

The comparisons between participants who remained in the study and participants who left the study in relation to the variables at baseline in each of the groups were performed using the Student's t test.

The comparisons between the groups in relation to the evolution (difference between the moments 24 months and baseline) of the outcomes (Lumbar BMD_0 to 24, Lumbar BMC_0 to 24, Total Body BMD_0 to 24, Total Body BMC_0 to 24, Subtotal BMD_0 to 24, Subtotal BMC_0 to 24, Fat Mass_0 to 24, Osteocalcin_0 to 24, BAP_0 to 24) were performed by fitting multiple linear regression including basal bone age, BMI, and Total body BMD as adjustment variables.

The groups were compared at baseline only among participants who completed 24 months, using the ANOVA model with fixed effects followed by Bonferroni.

Differences or relationships in the regression models were considered statistically significant if $P < 0.05$. Analyses were performed using SPSS 21 software.

.
