# Supplementary material for: Bone impact after two years of low-dose oral contraceptive use during adolescence
Source: PLoS One. 2023 Jun 8;18(6):e0285885. doi: 10.1371/journal.pone.0285885 (PMC10249826; doi:10.1371/journal.pone.0285885)
Supplement: S2 File — (PDF) [file pone.0285885.s005.pdf]

Trial Protocol

Portuguese Version

Anticoncepcionais hormonais orais de baixa dosagem na adolescência e repercussões sobre a massa óssea: dois anos de uso

Objetivo: Avaliar o comportamento do metabolismo ósseo de dois grupos de adolescentes saudáveis, usuárias de anticoncepcional hormonal oral (ACHO), ACHO 1: Etinilestradiol (EE) 20 µg / Desogestrel 150 µg ou ACHO 2: EE 30 µg / Drospirenona 3 mg, durante um período de dois anos de uso, comparativamente a um grupo-controle de adolescentes não-usuárias de ACHO.

Trata-se de um estudo longitudinal controlado não-randomizado (quase experimental) em que foram incluídas adolescentes do sexo feminino de 12 a 20 anos incompletos, saudáveis, voluntárias, que já apresentaram menarca, estadiamento de desenvolvimento puberal de Mamas M4 ou M5, de acordo com os critérios de Tanner, atendidas em ambulatórios das regiões de Assis, e avaliadas pela autora do presente estudo e, em Botucatu, as adolescentes matriculadas no ambulatório de Medicina de Adolescentes e no Ambulatório de Convênios - Adolescentes do Hospital das Clínicas da Faculdade de Medicina de Botucatu-UNESP.

As pacientes às quais não estava indicado nenhum método contraceptivo, por não terem vida sexual ativa, foram direcionadas para o grupo-controle. Todos os métodos contraceptivos utilizados pelos profissionais da saúde, para essa faixa etária, foram

expostos a todas as adolescentes, com indicação para uso de algum método. Aquelas que escolheram ACHO foram incluídas no estudo. Os ACHOs definidos para o estudo são combinações de EE 20 µg / Desogestrel 150 µg caracterizado como grupo ACHO 1 e EE 30µg / Drospirenona 3mg, como grupo ACHO 2.

Todas foram orientadas e estimuladas ao uso de dupla proteção para prevenção de IST mediante o uso do preservativo masculino concomitantemente ao uso do ACHO.

As adolescentes, submetidas a minuciosa anamnese, não eram tabagistas, etilistas, usuárias de drogas ou medicamentos que pudessem interferir no incremento da massa óssea, nem praticantes de modalidade esportiva extraescolar, exceto duas horas de aulas de Educação Física oferecidas na própria escola, e não apresentavam histórico prévio de uso de contraceptivos hormonais ou de gestação.

As adolescentes com histórico de prematuridade ou baixo peso ao nascimento, e as submetidas à terapia prolongada com corticóides ou que estivessem usando suplementação com cálcio e /ou ferro não foram não eram elegíveis. Adolescentes portadoras de: *diabetes mellitus*, desnutrição aguda ou crônica, doenças ósseas congênitas ou adquiridas, doenças gastrintestinais acompanhadas de má absorção, histórico de nefropatia, com ou sem insuficiência renal crônica, endocrinopatias, puberdade precoce ou atrasada, fibrose cística, doença celíaca, ou usuárias de medicamentos que, sabidamente, afetam o metabolismo ósseo de maneira negativa, como anticonvulsivantes, anticoagulantes, antirretrovirais e antiácidos com alumínio, não foram incluídas.

Para aquelas que não compareceram no dia agendado para realizarem sua densitometria ou coleta sanguínea, foram oferecidos novos agendamentos, em dias próximos aos definidos para realização dos exames. Caso não comparecessem nas três oportunidades oferecidas, para realização dos exames, ou se, os resultados obtidos em algum dos momentos de acompanhamento prospectivo, no caso, aos 12 meses com

finalização aos 24, estivessem incompletos, as adolescentes seriam incluídas nas análises estatísticas até o momento em que permanecessem em acompanhamento.

Algumas adolescentes dos grupos de ACHO, por motivos pessoais, desistiram do uso de contraceptivos hormonais orais, passando a utilizar outros métodos de contracepção, e, assim como adolescentes do grupo-controle, também foram excluídas da coleta de exames, por necessitarem de prescrição de algum método contraceptivo, o que invalidaria sua permanência no grupo-controle.

A todas foram oferecidos os mesmos cuidados prestados às que permaneceram no acompanhamento pelo tempo proposto e, de acordo com as normas dos Serviços de Saúde que utilizavam. A todas as adolescentes foi informado o número de *Whatsapp* das pesquisadoras e, a qualquer dúvida, poderiam fazer contato ou enviar mensagens.

As adolescentes assinaram um Termo de Assentimento, quando menores de 18 anos, e seus pais ou responsáveis foram orientados a assinar um Termo de Consentimento Livre e Esclarecido (TCLE) para autorizarem a participação na pesquisa. Aquelas com idade igual ou superior a 18 anos assinaram o TCLE.

Todas foram submetidas à consulta médica, a exames físicos gerais e exames específicos, foram-lhes coletados os dados antropométricos e feita avaliação dos caracteres sexuais secundários, através da inspeção visual das mamas e pelos pubianos, classificados segundo os critérios de Tanner.

As adolescentes participantes do estudo eram saudáveis, tinham estatura entre o 5º e o 95º percentil para cada faixa de idade e índice de massa corporal (IMC) variando entre o 5º e < 95º percentil, segundo curvas elaboradas pelo *Centers for Disease Control and Prevention* (2002).

A idade óssea (IO) para a avaliação do grau de maturação esquelética foi solicitada a todas as adolescentes. O método escolhido foi o de Greulich & Pyle (1959) chamado de

método GP, em que se faz a radiografia de mão e punho para ser posteriormente comparada com o Atlas. A interpretação foi realizada por um único avaliador habilitado (AST), mas sem saber previamente a que grupo a adolescente pertencia - cegamento do avaliador.

As adolescentes foram submetidas à avaliação da massa óssea no momento de iniciarem o uso do ACHO, após 12 e após 24 meses de seu uso, mediante um exame de densitometria óssea, em uma unidade de Densitometria Óssea por atenuação de raio X de dupla energia e com o uso de um aparelho Hologic QDR 4500 Discovery A (Hologic Inc., Bedford, MA). Para a adequada avaliação da massa óssea, foi utilizado um *software* pediátrico e os resultados do conteúdo ósseo foram expressos em gramas (g) e os da densidade, em g/cm^2. Foram mensuradas as regiões da coluna lombar entre L1-L4, densitometria de corpo total e subtotal, sem o segmento da cabeça. Todas as avaliações foram realizadas por apenas um profissional habilitado (AST) que, ao realizar o exame de densitometria, não foi informado (avaliador cego) se a adolescente estava ou não usando algum ACHO. Segundo atualizadas orientações da *International Society for Clinical Densitometry* (ISCD) de 2019, os relatórios DXA contêm as seguintes informações: idade do paciente, peso e altura, história médica relevante**, r**esultados do estágio de Tanner e da idade óssea.

Amostras de sangue foram coletadas, nos três momentos, por punção venosa e centrifugadas por 15 minutos a 1.500 *g* para a separação do soro; forma armazenadas a -70◦C até a análise dos biomarcadores FAO e OC.

A OC foi dosada pelo método Micro Vue Osteocalcin EIA (enzyme immuno assay) kit da QUIDEL® (USA, CA, San Diego), como indicador de “turnover” ósseo. O imunoensaio é um teste de ELISA competitivo, que quantifica somente a OC intacta e não detecta fragmentos de tecido ósseo reabsorvido. Para realização desse teste utlizaram-se microplacas de 96 alvéolos previamente tratadas com Osteocalcina e, sobre esses alvéolos

já tratados, foram adicionados 25µL de cada padrão reconstituído, controles e amostras de soro dos indivíduos testados. Em seguida, adicionaram-se 125µl de anticorpo anti-Osteocalcina, com posterior incubação de 120 minutos à temperatura ambiente e lavagem da placa com 30L de tampão de lavagem, por três vezes. Após lavagem da placa e retirada do anticorpo anti-Osteocalcina, foi–lhe adicionada a IgG anti- camundongo conjugada com a enzima fosfatase alcalina e, com posterior incubação, por 60 minutos, à temperatura ambiente. A placa foi lavada novamente, conforme descrito, e adicionado o substrato com p-Nitrofenil fosfato (pNPP) para o desenvolvimento de cor, por um período de 35-40 minutos. A reação foi bloqueada com NaOH 0,5N e a leitura realizada em leitor de Elisa TP Reader, Thermo Plate, no comprimento de onda de 405 nm. As concentrações de Osteocalcina nas amostras de soro foram obtidas, usando-se a fórmula $y= (A-D) / (1+ (x/C)^B)+D$. O intervalo de concentração utilizado para o teste foi de 2 a 32 ng/mL, com limite mínimo de detecção de 0,45 ng/mL, sendo determinado por três vezes o valor do desvio, para o padrão zero. O coeficiente de variação obtido para a precisão do ensaio descrito pelo fabricante foi de 5 a 10% para o intra-assay e 5 a 10% para o inter-assay.

A FAO foi dosada utilizando-se o Micro Vue BAP EIA (enzyme immuno assay) kit da QUIDEL® (USA, CA, San Diego), como indicador de atividade osteoblástica. O imunoensaio é um teste que utiliza um anticorpo monoclonal anti-FAO, como captura, e é adsorvido à superfície dos alvéolos da microplaca de 96 orifícios. Esse anticorpo monoclonal, que é adsorvido à placa, captura a FAO presente nas amostras de soro, padrão e controles, sendo possível determinar a atividade da FAO com o substrato p-Nitrofenil fosfato (pNPP). Para isso, um volume de 125µL de tampão de ensaio foi acrescido conjuntamente à placa, seguido de 20 µL de volume de soro, controles e padrão. Após a retirada das amostras de soro, padrão e controles, a placa foi, então, lavada quatro vezes com tampão de lavagem e foram adicionados 150 µL do pNPP. O desenvolvimento de cor ocorreu dentro de um período de 30-35 minutos à temperatura ambiente, a reação foi bloqueada com NaOH 0,5N e a leitura realizada em microleitor de Elisa TP Reader, Thermo Plate, em comprimento de

onda de 405 nm. As concentrações de FAO nas amostras de soro foram obtidas através da fórmula y= $A+Bx+Cx^2$. O intervalo de concentração utilizado para o teste e montagem da curva-padrão compreendeu concentrações entre 2 a 140 U/L. O limite mínimo de detecção para esse ensaio fornecido pelo fabricante era de 0,7U/L, sendo determinado por três vezes o valor do desvio para o padrão zero. O coeficiente de variação obtido para a precisão do ensaio era de 4,0 a 6,0% para o intra-assay e 5,0 a 8,0% para o inter-assay.

Para o cálculo amostral das adolescentes a serem incluídas em cada um dos grupos propostos, desfecho DMO, considerando-se um desvio-padrão semelhante entre os grupos e igual a 2, com erros tipo I e tipo II iguais a 0,05 e 0,20, respectivamente, estimou-se uma amostra de 37 adolescentes em cada grupo, o ACHO 1, o ACHO 2 e grupo-controle. Os cálculos foram obtidos através de pré-testes e estudos prévios realizados com adolescentes sob as mesmas condições do presente estudo, admitindo-se nível de significância de 5% para o intervalo de confiança de 95%, determinados para uma distribuição normal.

Análise estatística

Não houve violação significativa dos pressupostos teóricos de normalidade dos resíduos (por meio do teste de Shapiro-Wilk e histogramas) e homocedasticidade (por meio do teste Levene e dispersão entre resíduos e preditos dos modelos), corroborando os modelos adotados.

A comparação entre os grupos em relação as variáveis antropométricas, densitométricas e marcadores ósseos no momento basal e após 24 meses envolvendo todas as participantes selecionadas foi feita por modelo ANOVA de efeitos fixos seguido do teste de Bonferroni para comparações múltiplas.

A comparação entre participantes que permaneceram no estudo e participantes que saíram do estudo em relação as variáveis no momento basal em cada um dos grupos foi feita pelo teste t de Student.

A comparação entre os grupos em relação a evolução (diferença entre momentos 24 e basal) dos desfechos (DMO Lombar_0a24, CMO lombar_0a24, DMO Corpo Total_0a24, CMO Corpo Total _0a24, DMO SubTotal _0a24, CMO SubTotal _0a24, Massa gorda_0a24, Osteocalcina_0a24, FAO_0a24) foi feita ajustando regressão linear múltipla incluindo idade óssea basal, índice de massa corpórea e densidade mineral óssea do corpo total, todos no momento basal como variáveis de ajuste.

Os grupos foram comparados no momento basal somente entre as participantes que completaram 24 meses por meio de modelo ANOVA com efeitos fixos seguido de Bonferroni. As diferenças ou relações nos modelos de regressão foram consideradas estatisticamente significativas se $p < 0,05$. Análises foram feitas com o software SPSS 21.

Comitê de ética em pesquisa

O presente projeto foi apresentado ao CEP, em janeiro de 2016, quando de seu envio à FAPESP sob o número CAAE: 52928416.6.0000.5411 e foi aprovada uma emenda no ano de 2018 junto à Plataforma Brasil sob o número 2.766.807 para dar continuidade à coleta de dados.
