# Supplementary material for: Bone impact after two years of low-dose oral contraceptive use during adolescence
Source: PLoS One. 2023 Jun 8;18(6):e0285885. doi: 10.1371/journal.pone.0285885 (PMC10249826; doi:10.1371/journal.pone.0285885)
Supplement: Attachment — Submitted filename: 17_12_2021 Answers to Internal Staff.docx [file pone.0285885.s008.docx]

To

Mr. Michael Esguerra Alba

PLOS ONE

First, we are grateful for the opportunity to send a revised version of our paper “**Bone impact after two years of low-dose oral contraceptive use during adolescence**”. We have carefully read the comments to ensure that we have effectively responded to the suggestions and questions raised. Below are the comments made and, in sequence, our answers, highlighted in *blue*.

We would like to thank you for the possibility of re-presenting the study to PLOS ONE with the incorporation of the comments made by the in-house editorial staff.

1. Please note that you must upload a completed CONSORT flowchart as figure 1 of your manuscript and the CONSORT checklist as a supporting information file. Blank copies of these documents and information regarding CONSORT can be found via the following link: <http://www.consort-statement.org/>. If your clinical trial uses a non-randomized design, you may wish to submit a TREND checklist
   (<http://www.cdc.gov/trendstatement>), in place of the CONSORT checklist; a flowchart is still required.”

***Answer:*** Considering the study as a non-randomized clinical trial, the flowchart is attached as figure 1 of the manuscript and the TREND checklist is uploaded as a supporting information file.

2- Please upload a copy of your trial study protocol as a supporting information file. By the study protocol, we mean the complete and detailed plan for the conduct and analysis of the trial that the ethics committee approved before the trial began. Please send this in the original language. If this is in a language other than English, please also provide a translation. Please detail any deviations from this study protocol in the Methods section of your manuscript. Your study protocol will be made available to the editors and
reviewers, and will be published as supporting information with your manuscript if accepted for publication. (If you do not agree to this, we will not be able to publish your manuscript). If you have formally published a study protocol for your trial in a journal then you should cite this in your manuscript, but you still need to send us the original document.

***Answer:*** Find uploaded a copy of our trial study protocol as a supporting information file (original and translation file). The manuscript has not been previously published. The lead authors Lilian Rodrigues Orsolini and Prof. Tamara Beres Lederer Goldberg affirm that this manuscript is an honest, accurate, and transparent account of the study being reported; that no important aspects of the study have been omitted, and that no discrepancies from the study protocol have been made.

3- The in-house editorial staff feels that your study meets the World Health Organization definition of a clinical trial because it is a prospective study in which participants were assigned to Ethinylestradiol (EE)/Desogestrel or EE/Drospirenone over a period of two years  to investigate the effects on bones.

***Answer:*** Thank you for your comment, the alterations have been made.

Kind regards

Prof. Tamara Beres Lederer Goldberg M.D., Ph.D.

Lilian Rodrigues Orsolini, M.D., M.S.
