# Supplementary material for: Bone impact after two years of low-dose oral contraceptive use during adolescence
Source: PLoS One. 2023 Jun 8;18(6):e0285885. doi: 10.1371/journal.pone.0285885 (PMC10249826; doi:10.1371/journal.pone.0285885)
Supplement: Attachment — Submitted filename: Response to Reviewers.docx [file pone.0285885.s009.docx]

Botucatu, November 20th, 2022.

Dear Dr Tischer and Reviewers

We thank you for your important suggestions that helped improve our manuscript. Some of the suggestions were accepted, while others, although pertinent, have not been included in the text, in which case, an explanation of our point of view has been provided. Below, please find our replies to the suggestions of the reviewers. We again thank the reviewers for their important comments and the detailed appraisal of our manuscript.

We hope to have clarified all doubts and remain at your disposal for further clarifications.

Sincerely,

Prof. Tamara Goldberg, Lilian Rodrigues Orsolini, MSc and authors Review Comments to the Author

Reviewer #1: The present study is a quasi-experimental study evaluating the effect of a low-dose oral contraceptive on DXA in women aged 12 to 20 years. The authors found that oral	contraceptive	users	had	a	lower	BMD	when	compared	with	non-users.

The main study concerns are the number of subjects lost to the follow-up (About 44% were lost to the follow-up in the Ethinylestradiol (EE)/150 μg Desogestrel group), the broad age range (with a lower mean age in the control group), and some basal differences among the groups (the group with 30 μg EE/3 mg Drospirenone had a higher total BMD at the baseline).

**Answer:** Dear Reviewer, thank you for your comments, which have certainly enhanced our study. You are right in several of your questions, and we hope to have clarified them one by one below.

As we pointed out in the current article, accompanying adolescents is already an extremely arduous and laborious task, but rewarding, especially when we are offering protection, by promoting prevention and a better quality of life for this population.

As for your comment regarding the statistical differences evidenced for the total body BMD (Table 1), if we only used the sites recommended by the ISCD (2019) Pediatric (Lumbar and Subtotal), we would only include the results for the subtotal body (less head). We emphasize that for the subtotal BMD, no statistical differences were detected at baseline (Subtotal BMD (g/cm2) 0.905 ± 0.062 0.870 ± 0.063 0.893 ± 0.073 p= 0.063)

However, we decided to present all the results obtained, so that there would be no doubt as to **our scientific rigor**.

ISCD (2019) OFFICIAL POSITIONS: **DXA Interpretation and Reporting in Children and Adolescents**

*“DXA is the preferred method for assessing BMC and areal BMD. The posterior- anterior (PA) spine and total body less head (TBLH), are the preferred skeletal sites for performing BMC and areal BMD measurements in the majority of pediatric subjects. Other sites may be useful depending on the clinical need.”*

Unfortunately, the number of participants was not equal between the groups, due to the difficulty in maintaining COC use for a period of 2 years. Patient follow-up investigations suffered various losses owing to discontinuation of use, as well as non-attendance at all scheduled procedures. However, despite the difference in the number of participants between the groups, at the initial moment, the groups demonstrated homogeneity in the majority of the variables analyzed, which significantly minimized the bias in the follow-up of the outcome between groups.

Specific comments

1) The age range appears wide even though the Tanner stages are B4 to B5. How do the authors explain and justify this age range?

**Answer:** The beginning of sexual activity occurring more and more precociously is a reality in our service, reflecting a reality in the country, and we cannot ignore that we have accompanied 12 years old adolescents who are already mothers. Measures to prevent teenage pregnancy should be adopted and extended to all determinants, since the social, psychological, and biological consequences are inevitable and devastating. The prescription of oral hormonal contraceptives for adolescents is a relatively safe method, which is easy to reverse to fertility when discontinued, and well accepted by this age group, as well as not being used only for contraception. Although it interferes in the bone metabolism of adolescents, the deleterious effect on bone mass could be considered minor and possibly controllable when compared to the consequences of a pregnancy during this phase of life.

It should be remembered that one of the strict selection criteria is the non-previous use of hormonal contraceptives or pregnancy.

Since puberty can start from 8 years of age in girls and menarche occurs on average 2-3 years after the onset of pubertal development (around stage B4), we remind you that B4 or B5 breasts are considered components of late puberty ^1^. We emphasize that the average onset of puberty in Brazil occurs when girls are around 9.6 years of age, PHV at around 11.4 years of age, and menarche at 12.4 years of age^3,4^. In addition, some adult women may remain in the B4 stage of breasts (Tanner stage), not progressing to B5.

1. Marti. KA, Barbieri RL. Overview of the use of estrogen-progestin contraceptives. [www.uptodate.com,](http://www.uptodate.com/) Mar, 2018.
2. Colli AS. **Crescimento e desenvolvimento físico**. São Paulo (Ed.), Secretaria de Estado da Saúde de São Paulo. Comissão de Saúde do Adolescente. Adolescência e saúde., Paris Editorial, São Paulo (1988), pp. 43-57
3. Fortes CM, Goldberg TB, Kurokawa CS, Silva CC, Moretto MR, Biason TP, et al. Relationship between chronological and bone ages and pubertal stage of breasts with bone biomarkers and bone mineral density in adolescents. J Pediatr (Rio J). 2014;90:624-31.

2) How was chosen the combination of oral contraceptive? 20 μg Ethinylestradiol (EE)/150 μg Desogestrel (COC1 group) or 30 μg EE/3 mg Drospirenone (COC2 group)?

**Answer:** There is consensus among specialists regarding the prescription of COC, composed of increasingly lower doses of EE in order to reduce thromboembolic complications (4). Low‐estrogen COCs (less than 20 micrograms) have been attributed to more breakthrough bleeding and spotting and sub‐optimal cycle control impairs the acceptance of and adherence to the contraceptive, as most women identified bleeding irregularities as the primary reason for discontinuation (5).

Knowing that estrogen exerts a strong influence on the acquisition of bone mass for adults in pre-menopause and menopause and that contraceptive use among adolescents has become increasingly frequent and precocious, the present study hypothesized that a dose of Ethinylestradiol higher than 30 micrograms could be beneficial for bone health at this age, resulting in a greater increase in bone mass when compared to the lowest dose of 20 micrograms, however this hypothesis was not confirmed in the present study.

Another point to be highlighted is that the medicines were obtained free of charge and randomly provided to our patients, according to availability on the day of the consultation.

4. Agostino H, Di Meglio G. Low-dose Oral Contraceptives in Adolescents: How Low Can You Go? J Pediatr Adolesc Gynecol. 2010;23: 195–201. doi:10.1016/j.jpag.2009.11.001
5. Gallo MF, Nanda K, Grimes DA, Lopez LM, Schulz KF. 20 µg versus >20 µg estrogen combined oral contraceptives for contraception. Cochrane Database of Systematic Reviews 2013, Issue 8. Art. No.: CD003989. DOI: 10.1002/14651858.CD003989.pub5.
3) How Desogestrel compares with Drospirenone? Why have the authors chosen to use different progestogens?

**Answer:** Dear Reviewer, your question is excellent. We have answered part of your question in the previous question and will answer the rest below.

The progestins present in the composition of the COCs used, drospirenone and desogestrel, are known to act on androgen receptors by competitive inhibition, with this mechanism resulting in important antiandrogenic effects on bone metabolism, since testosterone plays a key role in bone mass acquisition. Hadji et al. found no effect on the fracture risk of users of contraceptives containing only progestin, demonstrating the preservation of bone mass. Thus, the effect of progestin on bone metabolism may be associated with its combination with the estrogen component, either 17β-estradiol or EE, and with the route of administration, either oral or transdermal (6). A study comparing the use of different progestins observed a greater reduction in the concentrations of bone markers in the drospirenone group compared to the gestodene group, a progestin of the same generation as desogestrel used in our study (7). A meta-analysis concluded that progesterone combined with estrogen has an antiresorptive effect on bone. *In vitro*, progesterone exerts a stimulatory effect on osteoblast differentiation at physiological concentrations and an inhibitory effect at pharmacological concentrations (8).

The paucity of studies on the influence of progestins present in COC formulations makes it difficult to distinguish which effects on bone metabolism are arising from progesterone and which from estrogen levels. In addition, the heterogeneity of study designs impairs comparisons of the hormonal effect of oral contraceptives on bone remodeling in adolescents. The studies on this topic identified in the databases differed in terms of the type of contraceptives, EE concentrations, and time of onset of contraceptive use during bone mass acquisition. If bone mass acquisition during puberty could be optimized, it is likely that adults and older adults would be less susceptible to the complications of osteoporosis, since a 10% increase in peak bone mass results in a delay of 13 years in the onset of this condition (6).

6. Hadji P, Colli E, Regidor P-A. Bone health in estrogen-free contraception. Osteoporos Int [Internet] 2019 Dec 24;30(12):2391–2400.
7. Nappi C, Di Spiezio Sardo A, Greco E, Tommaselli GA, Giordano E, Guida M. Effects of an oral contraceptive containing drospirenone on bone turnover and bone mineral density. Obstet Gynecol 2005;105(1):53–60.
8. Seifert-Klauss V, Prior JC. Progesterone and Bone: Actions Promoting Bone Health in Women. J Osteoporos [Internet] 2010;2010:1–18.
4) Why have the authors used the BMI percentile instead of SD?

**Answer:** In the case of adolescents, we can work very adequately and as recommended by the CDC using percentiles, remembering that above the 85th percentile of the BMI, adolescents are considered overweight, with overweight considered between the 85th percentile <95^th^, and above the 95th percentile the adolescents are considered obese.

However, we would ask you to note that the BMI Z scores are also presented in the

Tables.

In the present study, we used the following inclusion criteria: the adolescents participating in the study were healthy and had a height and body mass index (BMI) between the 5th and 95th percentile for each age group according to the Centers for Disease Control and Prevention growth charts (9).

9. Kuczmarski R, Ogden C, Guo S, Grummer-Strawn L, Flegal K, Mei Z.

2000 CDC Growth charts for the United States: Methods and development. Vital Heal Stat. 2002;11: 1–190.

5) Please give the definition for the variables: total and subtotal body BMD. Why was the ISCD position not followed?

**Answer:** Dear reviewer, as we stated with regard to your first comment (see above), we use the ISCD recommendations and also present other variables.

6) Statistica analysis – why was the DXA not adjusted by the estimated bone age? Or basal BMI and BMD? It could be performed using a generalized linear model.

**Answer:** Dear reviewer we agree with the question raised and present an analysis with the adjustments in S1 Table below. We have verified that the results obtained corroborate the results presented and described in the version sent in December, 2021.

**S1 Table. Multiple linear regression for the comparison of the evolution of the variables between the groups adjusted by basal bone age, BMI, and Total body BMD.**

| **Outcome: Lumbar BMD_0a24** | **b** |  | **IC95%** |  | ***p*** |
| --- | --- | --- | --- | --- | --- |
| Intercept | 0.25 | -0.01 |  | 0.50 | *0.062* |
| COC2 | -0.05 | -0.11 |  | 0.01 | *0.089* |
| COC1  Controls | -0.02  _0_a | -0.09 |  | 0.05 | *0.602* |
| Bone age 0 | -0.02 | -0.03 |  | -0.01 | *0.006* |
| BMI 0 | 0.00 | 0.00 |  | 0.01 | *0.329* |
| Total body BMD 0 | 0.04 | -0.24 |  | 0.31 | *0.793* |
| p-Shapiro Wilk for residuals = 0.584 ; weak heterocedasticity |  |  |  |  |  |
| **Outcome: Lumbar BMC_0a24** | **b** |  | **IC95%** |  | *p* |
| Intercept | 31.88 | 13.60 |  | 50.16 | *0.001* |
| COC2 | -5.61 | -9.61 |  | -1.61 | ***0.006*** |
| COC1  Controls | -3.60  _0_a | -8.58 |  | 1.38 | *0.157* |
| Bone age 0 | -1.46 | -2.39 | -0.52 | | *0.002* |
| BMI 0 | 0.02 | -0.37 | 0.41 | | *0.932* |
| Total body BMD 0 | -3.18 | -22.61 | 16.25 | | *0.749* |
| p-Shapiro Wilk for residuals = 0.821 ; non-heterocedasticity |  |  |  | |  |

| **Outcome: Total body BMD _0a24** | **b** | **I** | **C95%** |  | *p* |
| --- | --- | --- | --- | --- | --- |
| Intercept | 0.33 | 0.17 |  | 0.49 | *0.000* |
| COC2 | -0.10 | -0.14 |  | -0.07 | ***0.000*** |
| COC1  Controls | -0.11  _0_a | -0.16 |  | -0.07 | ***0.000*** |
| Bone age 0 | 0.00 | -0.01 |  | 0.01 | *0.632* |
| BMI 0 | 0.00 | 0.00 |  | 0.00 | *0.578* |
| Total body BMD 0 | -0.15 | -0.32 |  | 0.02 | *0.081* |
| p-Shapiro Wilk for residuals = 0.141 ; non-heterocedasticity |  |  |  |  |  |
| **Outcome: Total body BMC_0a24** | **b** | **I** | **C95%** |  | *P* |
| Intercept | 1,026.47 | 653.18 | 1,399.75 | | *0.000* |
| COC2 | -315.41 | -395.82 | -234.99 | | ***0.000*** |
| COC1 | -322.39 | -421.94 | -222.83 | | ***0.000*** |
| Controls | _0_a |  |  |  |  |
| Bone age 0 | -33.43 | -52.79 | -14.07 | | *0.001* |
| BMI 0 | 1.21 | -6.79 | 9.22 | | *0.766* |
| Total body BMD 0 | -170.27 | -569.41 | 228.88 | | *0.403* |
| p-Shapiro Wilk for residuals = 0.148 ; non-heterocedasticity |  |  |  | |  |
| **Outcome: Subtotal BMD_0a24** | **b** | **I** | **C95%** | | *P* |
| Intercept | 0.17 | 0.06 | 0.28 | | *0.002* |
| COC2 | -0.02 | -0.05 | 0.00 | | *0.054* |
| COC1 | -0.03 | -0.06 | -0.01 | | ***0.017*** |
| Controls | _0_a |  |  |  |  |
| Bone age 0 | -0.01 | -0.01 |  | 0.00 | *0.052* |
| BMI 0 | 0.00 | 0.00 |  | 0.00 | *0.340* |
| Total body BMD 0 | -0.07 | -0.18 |  | 0.05 | *0.239* |
| p-Shapiro Wilk for residuals = 0.282 ; non-heterocedasticity |  |  |  |  |  |
| **Outcome: Subtotal BMC_0a24** | **b** | **I** | **C95%** |  | *P* |
| Intercept | 347.24 | -122.83 | 817.31 | | *0.148* |
| COC2 | -101.88 | -203.14 | -0.61 | | ***0.049*** |
| COC1 | -77.97 | -203.34 | 47.39 | | *0.223* |
| Controls | _0_a |  |  |  |  |
| Bone age 0 | -16.16 | -40.53 | 8.22 | | *0.194* |
| BMI 0 | -0.24 | -10.33 | 9.84 | | *0.962* |
| Total body BMD 0 | 21.07 | -481.56 | 523.69 | | *0.935* |
| p-Shapiro Wilk for residuals = 0.006 ; non-heterocedasticity |  |  |  | |  |

| **Outcome: Fat mass_0a24** | **b** | **IC95%** | | | | *P* |
| --- | --- | --- | --- | --- | --- | --- |
| Intercept | 1,172.91 | -2,2437.17 | | 2,4782.99 | | *0.922* |
| COC2 | -61.80 | -5,013.56 | | 4,889.96 | | *0.980* |
| COC1  Controls | -661.70  _0_a | -6,564.09 | | 5,240.69 | | *0.826* |
| Bone age 0 | -839.89 | -1,972.13 | | 292.34 | | *0.146* |
| BMI 0 | 139.55 | -322.81 | | 601.90 | | *0.554* |
| Total body BMD 0 | 12,098.85 | -12,509.96 | | 36,707.66 | | *0.335* |
| p-Shapiro Wilk for residuals < 0.001 ; non-heterocedasticity |  |  | |  | |  |
| **Outcome: BAP_0a24** | **b** | **IC95%** | | | | ***P*** |
| Intercept | -216.86 | -293.35 | | | -140.37 | *0.000* |
| COC2 | -6.08 | -21.64 | | | 9.49 | *0.444* |
| COC1 | -0.71 | -19.08 | | | 17.67 | *0.940* |
| Controls | _0_a |  | |  |  |  |
| Bone age 0 | 8.02 | 4.35 | 11.68 | | | *0.000* |
| BMI 0 | 1.11 | -0.39 | 2.62 | | | *0.147* |
| Total body BMD 0 | 47.32 | -32.17 | 126.82 | | | *0.243* |
| p-Shapiro Wilk for residuals = 0.314 ; non-heterocedasticity |  |  |  | | |  |
| **Outcome: Osteocalcin_0a24** | **b** |  | **IC95%** | | | *P* |
| Intercept | -36.23 | -66.18 | -6.28 | | | *0.018* |
| COC2 | -0.90 | -6.88 | 5.09 | | | *0.768* |
| COC1 | 0.24 | -6.65 | 7.14 | | | *0.945* |
| Controls | _0_a |  |  |  |  |  |
| Bone age 0 | 2.58 | 1.14 | 4.02 | | | *0.000* |
| BMI 0 | 0.20 | -0.38 | 0.78 | | | *0.495* |
| Total body BMD 0 | -11.98 | -43.04 | 19.07 | | | *0.450* |
| p-Shapiro Wilk for residuals = 0.693 ; non-heterocedasticity |  |  |  | | |  |
| *Note:* Controls: adolescents who did not use oral contraceptives. |  |  |  | | |  |

COC1: adolescents receiving an oral contraceptive containing 20 μg EE/150 μg desogestrel. COC2: adolescents receiving an oral contraceptive containing 30 μg EE/3 mg drospirenone.

BMI: Body mass index; BMD: Bone mineral density;	BMC: Bone mineral content;	BAP: Bone alkaline phosphatase;

a: reference value for estimate b in the control group

7) Please move Table 3 and the results presented on lines 284 to 288 to the results section.

**Answer:** We appreciate the considerations. The changes have been made.

Reviewer #2: The manuscript addresses a potentially interesting topic. The collected data are original and rich of information. The employed statistical methods are rather basic and not fully suited for the data at hand. Some detailed comments follow.

1. The droput rate is rather relevant. It is well-known that the complete case analysis could be biased. I am wondering why missing-at-random or a missing-not-at-random assumptions are completely neglected. Please, provide support of the underlying modelling assumptions.

**Answer:** Dear reviewer, we understand the excellent remarks made by the reviewer. In fact, the distribution of losses can influence the results of a study. For this reason, we tried to assess the impact of the losses (Table 3) and, according to the assessments, it was shown that there was no significant difference between the analyzed variables of the participants who remained in the study compared to those who did not remain during the follow-up.

2. Results in table 3 are based on simple t-tests. However, even simple tests are based on quite strong assumptions. As the sample sizes are rather small, those assumptions are hardly tenable. Please, provide evidence that all the assumptions to ensure the reliability of the tests are met.

**Answer:** Dear reviewer, Table 3 does not present the primary results of the study, but only a comparison to assess the impact of the loss of participants during the follow-up period. However, we agree with the reviewer in being concerned with the theoretical assumptions of the tests, as well as with the concern of using regression models that enable the comparison between three groups, correcting for potential confounding variables. For this reason, we have created a new table, presented previously in question 6 by Reviewer 1 (S1 Table), which shows the results of the comparisons between the three groups through linear regression with normal response, together with verification of the quality of the adjusted models through the distribution of residues and homoscedasticity.

3. The data are longitudinal in nature. Maybe I miss something, but this fundamental data feature is neglected. Association between repeated measurements must be considered.

**Answer:** Dear reviewer, the longitudinal character has been incorporated in the analysis of the S1 Table, which compares the three groups in relation to the evolution of the variables studied.

4. Please, show data description, as in table 1, for complete cases only.

**Answer:** Dear reviewer, thank you for your comment and we agree with the question raised. We present an analysis with the adjustments for complete cases in S2 Table below. We verified that the data obtained corroborate the results presented and described in the version sent in December, 2021. The strict selection criteria contribute to the agreement between the analyses and results presented in Tables 1 and S2.

S2 Table

**S2Table. Comparison of anthropometric and densitometric variables and bone formation markers**

**at baseline between adolescents receiving low-dose oral contraceptives and the control group for**

**complete cases.**

|  | **Controls (n=19)** | | **COC1 (n=23)** | | **COC2 (n= 34)** | |  |  |
| --- | --- | --- | --- | --- | --- | --- | --- | --- |
|  | **Mean** | **SD** | **Mean** | **SD** | **Mean** | **SD** | ***P Value*** |  |
| Age(years) | 15.4 | 2.0 | 15.9 | 1.7 | 15.5 | 1.4 | 0.545 |  |
| Bone age (years) | 15.8 | 1.9 | 16.1 | 1.2 | 16.3 | 1.1 | 0.578 |  |
| Weight (kg) | 55.0 | 8.4 | 52.7 | 8.0 | 54.2 | 8.3 | 0.683 |  |
| Height (cm) | 161.2 | 5.0 | 159.9 | 6.5 | 157.7 | 6.9 | 0.142 |  |
| BMI (kg/m^2^) | 21.0 | 3.0 | 20.6 | 2.6 | 21.7 | 2.8 | 0.346 |  |
| Z-score for BMI | 0.2 | 0.8 | 0.0 | 0.8 | 0.4 | 0.7 | 0.138 |  |
| BMI (percentile) | 55.3 | 25.5 | 49.2 | 26.1 | 62.5 | 22.4 | 0.154 |  |
| Lumbar BMD (g/cm^2^) | 0.898 | 0.087 | 0.905 | 0.063 | 0.948 | 0.108 | 0.138 |  |
| Lumbar BMC (g) | 47.08 | 9.02 | 48.03 | 5.17 | 50.32 | 8.14 | 0.333 |  |
| Z-score for lumbar | -0.4 | 0.8 | -0.6 | 0.8 | -0.1 | 1.0 | 0.169 |  |
| Total body BMD (g/cm^2^) | 1.130 ^a^ | 0.073 | 0.974 ^b^ | 0.053 | 1.016 ^b^ | 0.070 | **0.000** |  |
| Total body BMC (g) | 1,982.04 | 277.09 | 1,783.17 | 205.59 | 1,815.40 | 249.53 | **0.037** |  |
| Z-score for total body | 1.4 ^a^ | 0.9 | -1.1 ^b^ | 0.6 | -0.4 ^c^ | 0.9 | **0.000** |  |
| Subtotal BMD (g/cm^2^) | 0.911 ^a^ | 0.051 | 0.852 ^b^ | 0.049 | 0.890 ^ab^ | 0.062 | **0.013** |  |
| Subtotal BMC (g) | 1,366.27 | 214.76 | 1,364.67 | 165.25 | 1,395.26 | 209.01 | 0.839 |  |
| Fat mass (g) | 18,666.3 | 4330.4 | 17,722.1 | 6610.5 | 17,903.0 | 5262.2 | 0.851 |  |
| Lean mass (g) | 34,590.2 | 4,822.7 | 36,724.1 | 4,442.7 | 35,093.6 | 7953.5 | 0.625 |  |
| Total body fat (%) | 33.8 | 4.3 | 29.9 | 4.0 | 31.6 | 5.1 | 0.058 |  |
| BAP (U/L) | 47.07 | 23.85 | 56.14 | 32.07 | 44.26 | 19.68 | 0.322 |  |
| Osteocalcin (ng/mL) | 16.66 a | 8.10 | 9.32 b | 5.15 | 10.97 b | 6.25 | **0.003** |  |
|  |  |  |  |  |  |  |  |  |

*Note:* Controls: adolescents who did not use oral contraceptives.

COC1: adolescents receiving an oral contraceptive containing 20 μg EE/150 μg desogestrel.

COC2: adolescents receiving an oral contraceptive containing 30 μg EE/3 mg drospirenone.

BMI: Body mass index; BMD: Bone mineral density; BMC: Bone mineral content; BAP: Bone alkaline phosphatase;

ANOVA for comparison of means between the three groups.

Different lowercase letters indicate significant differences between the three groups (p<0.05). Bonferroni test for multiple

comparisons between the three groups.

Total body BMC (g): no significant differences comparing groups using Bonferroni test

5. Please, provide evidence that "The assumptions of homoscedasticity and normality were tested using the Levene and Shapiro-Wilk tests, respectively, and the results showed normal distribution".

**Answer:** We appreciate the comments and therefore for the S1 Table models, the goodness of fit was verified using the Shapiro Wilk test for residuals and through the behavior of the residuals versus the predictive values of the adjusted model.

6. I am also wondering why, with such a rich dataset, a basic ANOVA or simple t-tests are considered only. Confounders may play a role and a regression analysis (for longitudinal data, taking into account missingness) may reveal interesting insights, currently swept under the carpet. Of course, model's assumptions must be checked carefully.

**Answer:** Dear reviewer, we agree and appreciate the comments. For this reason, new comparison analyses between the groups were carried out considering the suggested confounders and also the longitudinal character of the data, presented in S1 Table.

We appreciate all the comments and suggestions presented and hope that the answers have clarified your questions. We are available for further considerations.

Prof. Tamara Beres Lederer Goldberg M.D., Ph.D. Lilian Rodrigues Orsolini, M.D., M.S.
