# Supplementary material for: Bone impact after two years of low-dose oral contraceptive use during adolescence
Source: PLoS One. 2023 Jun 8;18(6):e0285885. doi: 10.1371/journal.pone.0285885 (PMC10249826; doi:10.1371/journal.pone.0285885)
Supplement: Attachment — Submitted filename: Response to reviewers.docx [file pone.0285885.s010.docx]

Botucatu, March 25th, 2023

Dear Dr Price and Reviewers

We thank you for your important suggestions that helped improve our manuscript. Some suggestions were accepted, and have been included in the text of the manuscript. A point-by-point response to the comments, including a detailed description of the changes made, is presented below. We again thank the reviewers for their important comments and the detailed appraisal of our manuscript. We hope to have clarified all doubts and remain at your disposal for further clarifications.

Sincerely,

Prof. Tamara Goldberg, Lilian Rodrigues Orsolini, MSc and authors

Editor’ comments to the Author

Comments:
1. Abstract: it would be more valuable to the reader to see some metric that describes the incorporation of bone mass and how it differs across groups, rather than just the p value. The p value tells you nothing about the relationship (except that it is statistically significant)
Abstract: “The three groups exhibited a reduction in the concentrations of bone formation markers, without a significant difference.” This is an oxymoron. If you do not see a significant difference in bone formation markers, than the bone formation markers across these three groups as statistically equivalent. To say “exhibited a reduction” is misleading and incorrect. In the discussion, you may wish to bring up statistical power, and that, had you enrolled more participants, you may have seen a statistical difference in the data. See also the results section and part of the discussion (page 14) – it appears as though you have presented these differences as significant when they are not.

**Answer:** Dear Reviewer, we appreciate your comments about our study and the changes have been made as suggested in the new version, to aid understanding.

Dear Reviewer, you are right that larger samples increase the power. However, the drop out reasons have been highlighted in the text and in Figure 1, and although the present study has some limitations, such as the loss to follow-up of some adolescents, an analysis of the power of the test for the **comparison of the variation in mean absolute values** for the outcomes lumbar spine, total body BMD, total body BMC, and Subtotal BMC between groups, using ANOVA with fixed effects, presented in Figure 2, showed estimated powers between 0.78 and 0.99, indicating relative adequacy of the analyzed sample size.

Dear Reviewer, thank you for your suggestion. For better understanding: ¨The mean concentrations of the bone markers (BAP and osteocalcin) decreased in the three groups. The reduction in the mean concentrations of the markers was lower in the COC2 group receiving 30 µg EE compared to the control and COC1 groups, but no statistically significant differences were found (p=0.686 and p=0.909, respectively). ¨

Considering the new Abstract, after your valuable considerations and also from the reviewers, we have included the new version below and in the manuscript:

**Objective**: Data regarding the use and effect of hormonal contraceptives on bone mass acquisition during adolescence are contradictory. The present study was designed to evaluate bone metabolism in two groups of healthy adolescents using combined oral contraceptives (COC). **Methods:** A total of 168 adolescents were recruited from 2014 to 2020 in a non-randomized clinical trial and divided into three groups. The COC1 group used 20 μg Ethinylestradiol (EE)/150 μg Desogestrel and the COC2 group used 30 μg EE/3 mg Drospirenone over a period of two years. These groups were compared to a control group of adolescent non-COC users. The adolescents were submitted to bone densitometry by dual-energy X-ray absorptiometry and measurement of bone biomarkers, bone alkaline phosphatase (BAP), and osteocalcin (OC) at baseline and 24 months after inclusion in the study. The three groups studied were compared at the different time points by ANOVA, followed by Bonferroni’s multiple comparison test. **Results**: Incorporation of bone mass was greater in non-users at all sites analyzed (4.85 g in lumbar Bone mineral content (BMC)) when compared to adolescents of the COC1 and COC2 groups, with a respective increase of 2.15 g and loss of 0.43g in lumbar BMC (P=0.001). When comparing subtotal BMC, the control increased 100.83 g, COC 1 increased 21.46 g, and COC 2 presented a reduction of 1.47 g (P= 0.005). The values of bone markers after 24 months are similar for BAP, being 30.51 U/L (± 11.6) for the control group, 34.95 U/L (± 10.8) for COC1, and 30.29 U/L for COC 2 (± 11.5) (P = 0.377). However, when we analyzed OC, we observed for control, COC 1, and COC 2 groups, respectively, 13.59 ng/mL (± 7.3), 6.44 ng/mL (± 4.6), and 9.48 ng/mL (± 5.9), with P =0.003. Despite loss to follow-up occurring in the three groups, there were no significant differences between the variables in adolescents at baseline who remained in the study during the 24-month follow-up and those who were excluded or lost to follow-up. **Conclusion**: Bone mass acquisition was compromised in healthy adolescents using combined hormonal contraceptives when compared to controls. This negative impact seems to be more pronounced in the group that used contraceptives containing 30 μg EE.

CLINICAL TRIAL REGISTRATION: <http://www.ensaiosclinicos.gov.br>, RBR-5h9b3c

“Low-dose combined oral contraceptive use is associated with lower bone mass in adolescents”.

**Keywords**: adolescents, bone mineral density, bone formation, bone remodeling, contraception

2.Abstract: no mention of the loss to follow up. This is an important point, I would add a sentence to clarify the high rate of attrition, and that this varied by group. In your reply to the comments, you note that those who dropped out did not differ from those who remained on study (am I interpreting this correctly? I’m referring to your comment “despite the difference in the number of participants between the groups, at the initial moment, the groups demonstrated homogeneity in the majority of the variables analyzed”). Is this covered adequately in your results section?

**Answer:** Thank you for your suggestion. A paragraph has been added to the abstract on the similarity between adolescents at baseline who remained in the study during the 24-month follow-up and those who were excluded or lost to follow-up (see question 1).

Yes, you are totally correctly in your interpretation.

3. Abstract, conclusions: As a clinician, I would want to know how big of an effect might I see with my patients who wanted to start birth control. As a public health / policy maker, I would want to weigh this against the value of effective birth control. Given your differential loss to follow up, I think it is safer to claim that there may be differences across the groups, but that your research does not definitively show this.

**Answer:** The study is not intended to harm the sexual and reproductive health of adolescents. It intends to contribute to the literature to assess whether there would be a better hormonal contraceptive option when considering adolescents, but many variables are involved in choosing a contraceptive method, from its effectiveness to adverse effects, non-contraceptive benefits, and adherence to use. We know that the quarterly injectable contraceptive presents a warning regarding the impact on bone mass during use and concern regarding the age group of adolescence. However, there is no absolute contraindication, only the recommendation regarding the possibility of choosing another option, if possible, without jeopardizing the prevention of teenage pregnancy. Our study seeks to contribute to the choice of the most appropriate contraceptive in terms of effectiveness and less adverse effects for the adolescent. Therefore, the results do not indicate an absolute contraindication, only an alert regarding possible prevention when the focus is on bone mass. However, more research is needed to make this conclusion and recommendations.

4. Materials and Methods: You note how the control group was assigned at the bottom of page 4; how were the two other groups assigned?

**Answer:** Unfortunately, the groups were not randomized. They were selected according to the demand at the adolescent outpatient clinic following strict inclusion criteria, with the adolescent who did not need contraception being defined as a control and for girls with an indication, all different contraceptive methods appropriate for this age group were presented by the health professionals and those who chose to use oral contraceptives were included in the study, being referred to COC 1 or COC 2. The medicines were obtained free of charge and provided to our patients, according to availability on the day of the consultation.

5. Materials and Methods: How do you define lost to follow up? When they stopped taking oral contraception? When they stopped attending study visits?
Methods, Statistical Analysis: Please define your outcome. How do you analyze participants who stay on study, but who stop (or switch) contraceptives?

**Answer:** Dear Reviewer, this information is present in figure 1, the adolescents lost to follow up were those who stopped taking oral contraception or changed the route of administration or presentation, and also who changed their address and stopped attending the visits, or the adolescents who started intense physical activity. All adolescents were evaluated at intervals of 3 months during the proposed follow-up period of two years, to asses those who remained or withdrew from participating in the study for the reasons indicated. On this occasion, the continuity of use of the prescribed COC was analyzed, as well as the permanence of the adolescent in the study according to the strict criteria proposed for their inclusion, including controls, who were not using contraceptives, who were involved in interpersonal relationships, and in activities that led to their exclusion from the study.

The outcomes considered for the present study were the evolutions between the densitometric parameters in the evaluated sites and biochemical markers between baseline and 24 months (S3 Table, S1Table, Figure 2).

These participants were excluded because they were no longer under the contraceptive effect to assess the desired outcome. The same care was provided to all adolescents regardless of who continued or not in the follow-up for the proposed period, according to the guidelines of the health services they attended.

6. Methods, Statistical Analysis: Kindly add a little more detail on how you assess any bias due to loss to follow up (e.g., did you compare those retained and analyzed with those enrolled to see if they differed by any variables that might confound or bias your results?). There’s nothing on this here, and I fear it may seriously compromise your modeling.

**Answer:** Thank you for your question and concern. This is a good observation. If we analyze the results presented in Table 1 and Table S2, we can verify the homogeneity between the groups.

7. Last sentence of results “This fact reinforces the results obtained despite a high percentage of losses in the groups after 24 months of follow-up, ruling out interference or bias in the results”. This is discussion, I recommend noting this there, as a strength of your study.

**Answer:** Thank you for your suggestion, the alterations have been made.

8. Discussion: you observed a statistically significant difference. Do you also feel this is a clinically significant difference? I feel this is a very important element of your paper that gets limited discussion. The final sentence of the first paragraph in the discussion suggests that may be the case, but I’d like to see more clarity on this from the authors.

**Answer:** Dear Reviewer, we appreciate your comments. The study seeks to contribute to the choice of the most appropriate contraceptive in terms of effectiveness and less side effects for the adolescent. Therefore, our results, although statistically significant, do not indicate a contraindication, only an alert regarding possible prevention when the focus is bone mass. See question 3.

[Note: HTML markup is below. Please do not edit.]

Reviewers' comments:

Reviewer's Responses to Questions

**Comments to the Author**

1. If the authors have adequately addressed your comments raised in a previous round of review and you feel that this manuscript is now acceptable for publication, you may indicate that here to bypass the “Comments to the Author” section, enter your conflict of interest statement in the “Confidential to Editor” section, and submit your "Accept" recommendation.

Reviewer #1: All comments have been addressed

Reviewer #2: (No Response)

2. Is the manuscript technically sound, and do the data support the conclusions?

The manuscript must describe a technically sound piece of scientific research with data that supports the conclusions. Experiments must have been conducted rigorously, with appropriate controls, replication, and sample sizes. The conclusions must be drawn appropriately based on the data presented.

Reviewer #1: Yes

Reviewer #2: Partly

3. Has the statistical analysis been performed appropriately and rigorously?

Reviewer #1: Yes

Reviewer #2: No

4. Have the authors made all data underlying the findings in their manuscript fully available?

The [PLOS Data policy](http://www.plosone.org/static/policies.action#sharing) requires authors to make all data underlying the findings described in their manuscript fully available without restriction, with rare exception (please refer to the Data Availability Statement in the manuscript PDF file). The data should be provided as part of the manuscript or its supporting information, or deposited to a public repository. For example, in addition to summary statistics, the data points behind means, medians and variance measures should be available. If there are restrictions on publicly sharing data—e.g. participant privacy or use of data from a third party—those must be specified.

Reviewer #1: Yes

Reviewer #2: No

5. Is the manuscript presented in an intelligible fashion and written in standard English?

PLOS ONE does not copyedit accepted manuscripts, so the language in submitted articles must be clear, correct, and unambiguous. Any typographical or grammatical errors should be corrected at revision, so please note any specific errors here.

Reviewer #1: Yes

Reviewer #2: Yes

6. Review Comments to the Author

Please use the space provided to explain your answers to the questions above. You may also include additional comments for the author, including concerns about dual publication, research ethics, or publication ethics. (Please upload your review as an attachment if it exceeds 20,000 characters)

Reviewer #1: I thank the authors for the through responses. All my comments have been addressed. I believe that the manuscript is now much improved.

**Answer:** Thank you for all your comments.

Reviewer #2: Though the authors provided some answers to my questions, there is a general lack of statistical knowledge and this strongly limits the work.
Two aspects still deserve more attention: the missing data mechansim and the longitudinal structure of the data.
1. Missingness cannot be "tested" by comparing the covariates between the retained and the dropout groups. The missing data mechanism refers to the main outcomes, and there is a wide literature on the effects of missing-not-at-random mechanisms. As it stands, the modelling approach may lead to biased estimates.

**Answer:** Dear Reviewer 2, we appreciate the reviewer's relevant observation. A simulation study demonstrates that there is little gain from performing multiple imputation compared to a full-case analysis in the presence of up to 50% missing data, when the data are Missing Completely at Random (MCAR) (Karahalios et al., 2013). Our database has 54% missing participants and about 35% missing data, and except for one specific variable at 24 months, our database at baseline and at 24 months did not reject the null hypothesis that the data are MCAR (p > 0.05; Little's Missing Completely at Random Test (MCAR); naniar R software package). For this reason, we believe that, if it exists, the effect of losses on estimation biases in our data is not relevant.

Reference: Karahalios, A., Baglietto, L., Lee, K.J. *et al.* The impact of missing data on analyses of a time-dependent exposure in a longitudinal cohort: a simulation study. Emerg Themes Epidemiol 2013: **10**, 6 (2013). <https://doi.org/10.1186/1742-7622-10-6>

2. " the longitudinal character has been incorporated in the analysis of the S1 Table, which compares the three groups in relation to the evolution of the variables studied". It is rather unclear how the longitudinal structure is accounted for. Did you consider a random effects model, a fixed effect or...? How did you estimate the parameters? Did you test the underlying modelling assumptions? Is there any time-dependence which needs to be considered?

**Answer:** We agree with the reviewers, since repeated measures can be analyzed in several ways, such as, for example, considering the various measurements as a multivariate vector, incorporating the autocorrelation structure, or even considering a simplified outcome by subtracting values obtained at two moments, creating a variable of evolution. We opted for this last described method because our objective was not to investigate the main effect of time and the interaction between groups and time through an ANOVA model with repeated measures. In this way, we did not need a model whose systematic component was capable of translating these questions. We were only interested in comparing the groups in relation to the evolution captured by the difference between the moments. The model adopted assumes a fixed effect. The parameters were estimated by least squares. Therefore, we opted for a technically accepted strategy, which eliminates the autocorrelation of the observation times, which meets our interests, is easy to understand, and is well accepted by clinicians. We thank the reviewers for their considerations and hope that we have clarified the questions raised by reviewer 2.

3. "The assumptions of homoscedasticity and normality were tested using the Levene and Shapiro-Wilk tests, respectively, and the results showed normal distribution"." Please, provide graphical evidence of this result.

**Answer:** Dear Reviewer, graphics have been provided, and are shown below.

The histograms of the residuals and the scatterplots between residuals and predictions of the adjusted ANOVA models do not suggest any gross violation of normality and homoscedasticity that would justify a replacement of the model used for data analysis.

**Gross residue BMCL 0-24**

**Frequency**

**Gross residue BMDL 0-24**

**Frequency**

**Gross residue BAP 0-24**

**Frequency**

**Gross residue OC 0-24**

**Frequency**

**Gross residue Subtotal BMD 0-24**

**Frequency**

**Gross residue Subtotal BMC 0-24**

**Frequency**

**Response mean predicted value	BMCL 0-24**

**Gross residue BMCL 0-24**

**Response mean predicted value	BMDL 0-24**

**Gross residue BMDL 0-24**

**Response mean predicted value	BAP 0-24**

**Gross residue BAP 0-24**

**Response mean predicted value	OC 0-24**

**Gross residue	OC 0-24**

**Gross residue Subtotal BMD 0-24**

**Response mean predicted value Subtotal BMD 0-24**

**Gross residue Subtotal BMC 0-24**

**Response mean predicted value Subtotal BMC 0-24**

**NOTE: The word “media” (term in portuguese) translated as mean**

7. PLOS authors have the option to publish the peer review history of their article ([what does this mean?](https://journals.plos.org/plosone/s/editorial-and-peer-review-process#loc-peer-review-history)). If published, this will include your full peer review and any attached files.


If you choose “no”, your identity will remain anonymous but your review may still be made public.


**Do you want your identity to be public for this peer review?** For information about this choice, including consent withdrawal, please see our [Privacy Policy](https://www.plos.org/privacy-policy).

Reviewer #1: No

Reviewer #2: No
